# An Account of the Efficacy of Vitriolic Æther, in Removing the Gout in the Stomach

**Published:** 1785

**Authors:** 


VI. *An account of the efficacy of Vitriolic Æther, in removing the gout in the stomach.* *By* James Lind, *M. D. F. R. S., Physician at Windsor, and Fellow of the Royal College of Physicians* *at*

*at Edinburgh. Communicated to Dr.* Simmons, *by Sir* Jofeph Banks, *Bart. P. R. S.*

I HAVE lately made trial of a medicine, which, in five different patients, has inftantly removed the gout in the ftomach, and in fome of them repeatedly. The fuccefs in every inftance where I have ufed it is fo flattering, that I am inclined to believe it a pretty certain, if not an infallible remedy for that purpofe.

The medicine alluded to is Vitriolic Æther; a tea-fpoonful of it in a glafs of water (or, as I commonly prefcribe it, in an ounce of camphorated julep, with half an ounce of fimple peppermint water) has removed the complaint in an inftant, when ardent fpirits, opium, and other medicines commonly ufed, had proved ineffectual; and I have found it equally efficacious, where no other medicine had preceded the exhibition of the Æther. As this practice, though mentioned by fome writers, feems to be not generally known, I beg to trouble you to communicate it to the public, for the benefit of thofe who may be afflicted with that dangerous and painful complaint.

*Windfor,*
*Feb.* 23, 1785.

VII. *An*

